# Management of oro-antral fistula: Two case reports and review

**DOI:** 10.1016/j.amsu.2021.102817

**Published:** 2021-09-04

**Authors:** Samir Mainassara Chekaraou, Laila Benjelloun, Karima EL Harti

**Affiliations:** Oral Surgery Resident/faculty of Dental Medicine-Rabat/ Mohammed V University of Rabat, Morocco

**Keywords:** Oro-antral fistula, Sinusitis, Surgical management, Buccal fat pad

## Abstract

Oro-antral Communication is an unnatural communication between the oral cavity and maxillary sinus and when it fails to close spontaneously, it remains patent and is epithelialized so that oro-antral fistula develops. It is a common occurrence following removal of maxillary premolars and molars because of anatomic proximity of root apices of these teeth and maxillary antrum. Signs and symptoms of oro-antral fistula varies from free escape of fluids, pain, pus leakage, voice alteration, to pan-sinusitis. Several surgical options exist for its management, in particular the buccal fat pad technique, which has proved to be an effective and a reliable technique. We report in this article two-succefull cases of oro-antral fistula managed with buccal fat pad.

## 1 Introduction

Oro-antral Communication (OAC) is an abnormal communication between the oral cavity and maxillary sinus and when it fails to close spontaneously, it remains patent and is epithelialized so that oro-antral fistula (OAF) develops [1]. This epithelialization usually occurs when the perforation persists for at least 48–72 hours [2].

These complications occur most commonly during extraction of upper molar and premolar teeth (48%). The major reason is the anatomic proximity or projection of the roots within the maxillary sinus. Other causes of OAC/OAF include tuberosity fracture, dentoalveolar/periapical infections of molars, implant dislodgement into maxillary sinus, trauma (7.5%), presence of maxillary cysts or tumors (18.5%), osteoradionecrosis, flap necrosis, dehiscence following implant failure and sometimes as a complication of the Caldwell-Luc procedure [3]. These situations can lead to maxillary sinus pathological conditions, which can prevent the resolution of the case.

There are many techniques for the closure of oroantral communication including buccal or palatal alveolar flaps and their modifications, various alloplastic materials like gold foil, gold plate, soft polymethylmethacrylate and lyophilized collagen, autogenous bone grafts were also used. The choice of technique depends on the size, the localization, and seniority of the lesion, but also on the surgeon's experience [4].

Among other available methods, the pedicled fat pad is a simple and reliable flap for the treatment of these defects.

Here we present two cases report treated successfully with the buccal fat pad (BFP) technique.

This case report has been reported in line with the SCARE Criteria [5].

## 2 Patient and observation

### 2.1 Case 1

A 57-year-old patient referred by his general dentist to our oral surgery department for air and pus leakage in the oral cavity. The patient had a well-balanced diabetes under treatment. On questioning, the patient reported a history of dental extraction eight months before.

The extraoral examination had no particularity. At the intraoral examination, we noted a bad oral hygiene with the absence of 16, 26, 35, 46 and 47. The mucosa around the site of 26 was normal with the presence of fistula detectable with a gutta-percha cone.

**Fig. 1:**
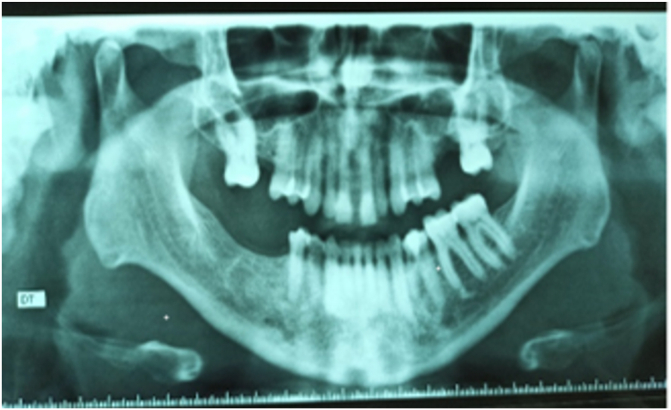
Panoramic radiograph showing a bone defect in 26 communicating the maxillary sinus with the oral cavity.

On the panoramic radiograph, a bone defect was noted making the left sinus communicate with the oral cavity (Fig. 1). The CT Scan confirmed the defect and showed a slight thickening of the sinus mucosa.

Considering all these data, the patient was put on antibiotics (amoxicillin/clavulanic acid for 10 days) and a nasal decongestant to treat sinusitis. A surgery with BFP was decided to close the oro-antral fistula.

Under local anesthesia, an intrasulcular incision going from 28 to 25 with mesial discharge allowed the lifting of a mucoperisoteal flap and the exposure of the fistula and bone defect A silky cleansing of the site was performed with elimination of the fistulous cord. A saline rinse to cleanse the sinus was also performed.

**Fig. 2:**
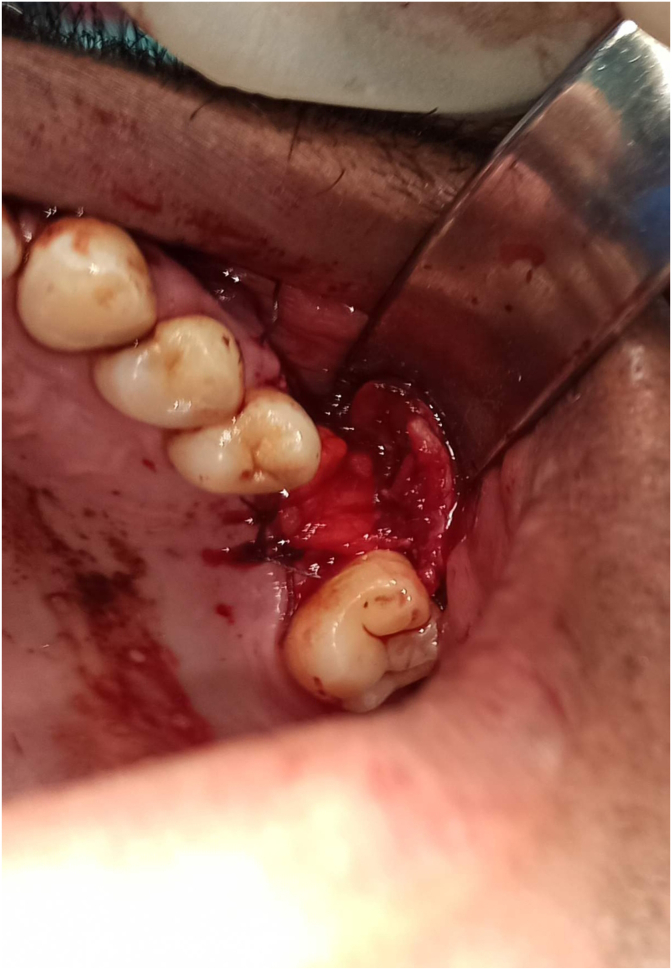
Intraoral view showing the traction and suturing of BFP at the site.

A horizontal incision at the level of the periosteum opposite to 28 was made to gain access to the buccal fat ball, which was dragged into the alveolar defect and then sutured on the bone defect (Fig. 2). Hermetic sutures were performed to reposition the flap to its initial position covering the BFP.

Antibiotics were continued for 10 days, along with prednisolone 60 mg/day for 5 days, paracetamol and nasal decongestant.

**Fig. 3:**
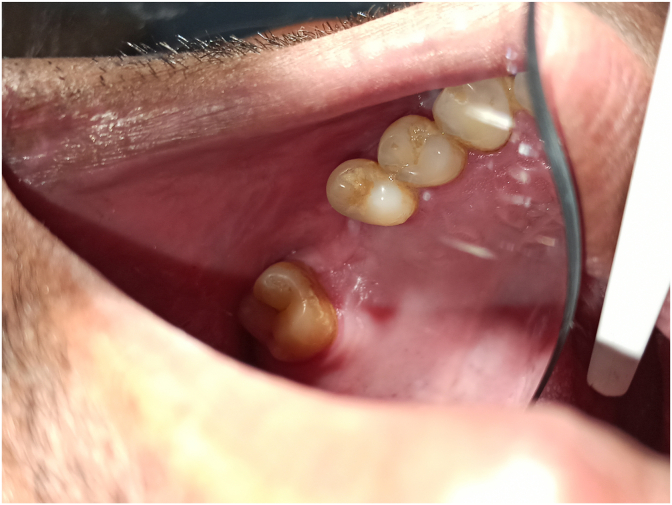
Intraoral view showing complete healing of the site after 6 months.

The patient was seen again after 10 days. Healing was uneventful with closure of the OAF. At six months, good healing was noted with a return to normal (Fig. 3).

### 2.2 Case 2

A patient aged 45 presented with lack of healing and air leakage from the extraction site of the first left maxillary molar, one month before extraction.

**Fig. 4:**
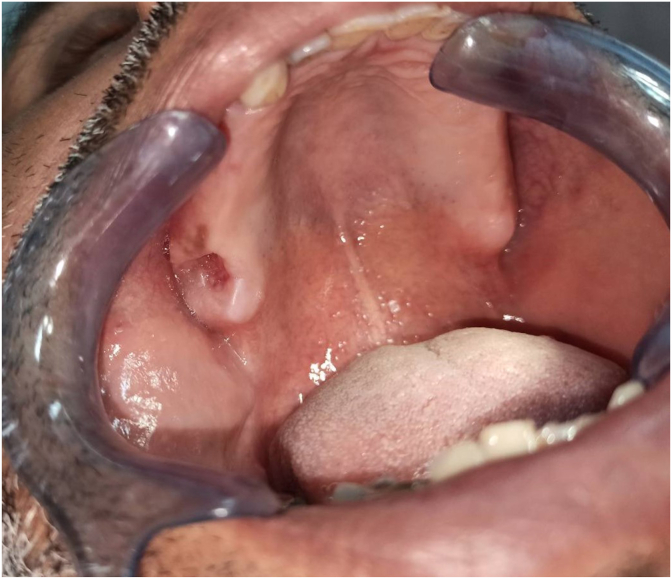
Intraoral view showing the site of communication.

On intraoral examination, an empty tooth socket was noted (Fig. 4).

**Fig. 5:**
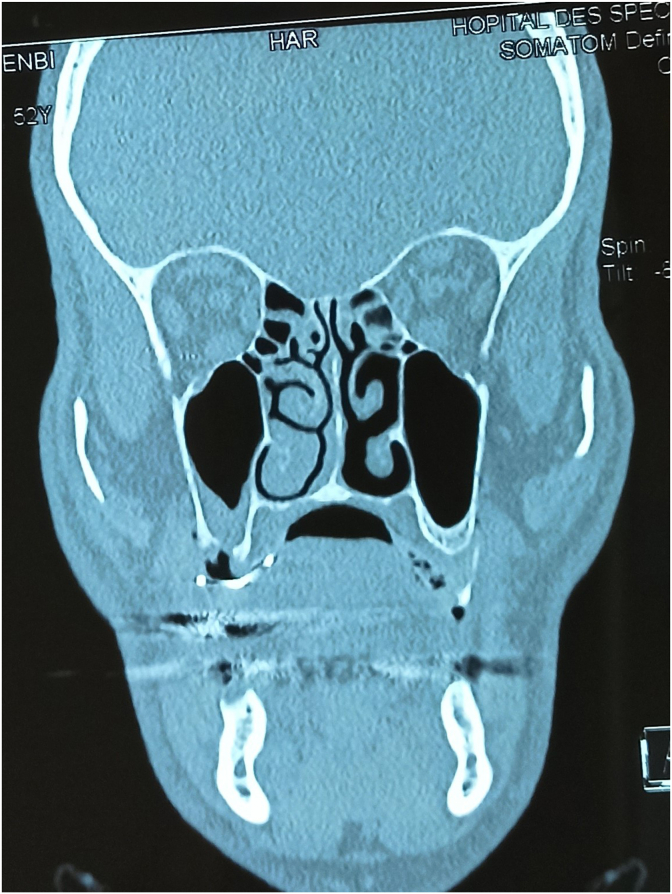
CT scan showing a thickening of the right sinus mucosa, poor aeration of the nasal meatus.

On the panoramic x-ray, a small bone defect was noticed making the oral cavity communicate with the right maxillary sinus. The CT scan showed a thickening of the right sinus mucosa, poor aeration of the nasal meatus and opacification of the ethmoidal air cells (Fig. 5).

**Fig. 6:**
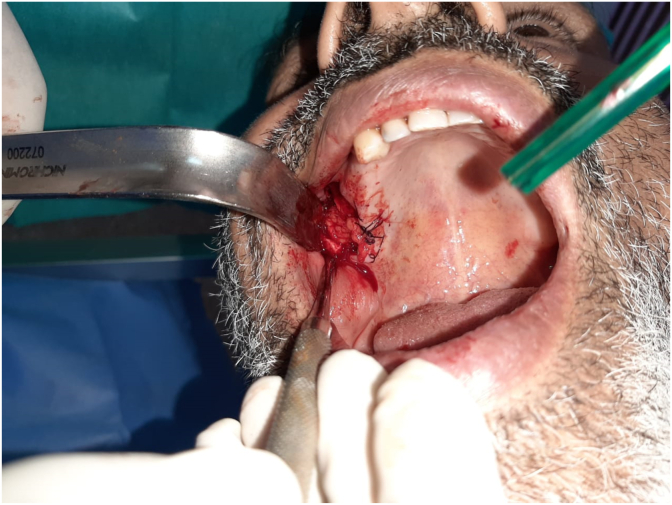
Intraoral view showing dissection and suture of the BFP at the site.

Therapeutic intervention and follow up: The patient was put on antibiotics (amoxicillin/clavulanic acid) for 10 days and surgical management with BFP under local anesthesia was decided in the following days to close the oro-antral communication. After the mucoperiosteal flap elevation, a large bone defect was revealed. The bone defect was closed with buccal fat pad after removing the fistula and rinsing the sinus with saline water. Hermetic sutures were used to suture the BFP over the bone defect (Fig. 6) and the repositioned flap.

Good healing was noted after 10 days and 6 months, without any complications.

## 3 Discussion

Oro-antral communications, or perforations that connect the mouth and the sinus, are commonly seen in clinical dental practice, especially after extractions of maxillary posterior teeth. According to the literature, the incidence of OAC has been reported to be as high as 11% [6,7]. Extraction of the palatal root of the maxillary first molar most often contributes to its formation **as in our case** [8].

If OAF is suspected, a thorough clinical and radiological examination should be proceeded. OAF acts as a pathway for bacterial and fungal penetration leading to maxillary sinusitis, or pan-sinusitis in 60% of cases [9,10]. Signs and symptoms may be acute or chronic. Acute symptoms include epistaxis, fluid or air passage through OAC/OAF, pain, voice alteration. Chronic symptoms include pain, free escape of fluids as in our cases, antral polyps, postnasal drip, dysgeusia, voice alterations, earache and mucopurulent nasal discharge [3].

Radiographic exams such as panoramic view allow us to see an alveolar defect and Waters’ view to see maxillary sinus infection. The communication between the oral cavity and maxillary sinus can be confirmed with Cone beam computed tomography or CT scan. They also permit to note a thickening of the sinus mucosa or its opacification, the aeration of the nasal meatus or the pathological state of the ethmoidal air cells or other sinuses.

Closure of the OAF is very important to prevent any food or saliva accumulation that can cause sinus contamination leading to infection, impaired healing and chronic sinusitis [10]. However, proper infection control must be performed prior to surgical closure of the fistula to prevent exacerbation of the infection and to permit the resolution of the case. In the case of patients with sinus infection, amoxicillin/clavulanic acid 1 g/125 mg three times per day for 10–14 days, nasal decongestants, and nonsteroidal anti-inflammatory drugs can be prescribed to manage sinusitis. The conduct of routine sinus irrigation could be helpful alongside the use of these medications.

Meanwhile, patients with chronic sinus disease that doesn't respond to medications will require surgical intervention such as endoscopic sinus surgery or the Caldwell–Luc procedure [11].

Closure of OAF should be based upon certain factors that not only influence the ultimate outcome of surgical closure but also post closure rehabilitation [12]. Some factors are the seniority and the Size of the defect. Usually an OAF <2mm in diameter closes spontaneously but when there is more than >3–4 mm defect, opening persists and requires closure [13].

Amongst the known techniques for closure of OAF are buccal advancement flaps, palatal advancement flaps, rotational advancement flaps, hinged flaps, island flaps, and buccal fat pad [14]. In our reported cases, given the size (>5 mm) and the seniority of the communication, the BFP technique was chosen.

It has been a popular method for the closure of oro-antral communications, as a single layer (Stajeic), with free skin grafts (Egyedi) or even covered by lyophilized porcine dermis (Fujimura). Heister [1,15] first described the anatomy of buccal fat pad in 1732 and in 1801 Bichat [1,15] verified its fatty histology. Egyedi, first reported its use as a pedicled graft [1,16].

The BFP consists of a main body and four extensions: buccal, pterygoid, superficial and deep temporal. The body is centrally positioned. The buccal extension lies superficially within the cheek, and the pterygoid and temporal extension are more deeply situated [16].

The surgical technique consists of a circular incision with 3mm margins, made around the OAF, epithelial tract and inflammatory tissue within the opening are completely removed. Two divergent cuts are then made from each end of the circular incision extending into the vestibule. The trapezoidal buccal mucoperiosteal flap is then reflected from the alveolar process and the lateral wall of the maxilla. The BFP is exposed through 1cm long vertical or horizontal incision in the reflected periosteum posterior to the zygomatic buttress. The BFP is dissected and gently advanced into the bony defect and sutured to the palatal mucosa without tension. Finally, the mucoperiosteal flap is replaced in its original position, and sutures are placed between BFP and the buccal flap so that a part of BFP is exposed into oral cavity. Epithelization of the exposed fat tissue occurs between 2 and 4 weeks postoperatively. The superficial layer of fat tissue becomes replaced by granulation tissue and is finally covered with stratified squamous epithelium migrating from the margin of the gingival [17]. The BFP can also be covered with the mucoperiosteal flap.

Regular post-operative follow-up of the patient is recommended for up to one year to avoid surgical failure and recurrence. Perfect healing was noted in our cases after more than six months of follow-up.

This technique presents many advantages, which are high success rate, wide applicability, simple procedure, done under local anesthesia, no additional removal of bone or tooth, low rate of complications, decrease risk of infections, can easily be trimmed to appropriate shape and no loss of sulcus depth. Its disadvantages are single use, the possibility of postoperative trismus, limited use for small and mid-sized defects and no rigid support [18].

## 4 Conclusion

Oral-antral fistula are frequent complications in dentistry. Their diagnoses require careful clinical and radiological examination. An early closure of post-chirurgical AOC is the best way to prevent AOF and sinus complications. Buccal fat pad represents a reliable treatment option that is easy and predictable for the closure of AOF.

## Funding source

No source has funded this manuscript.

## Ethical approval

All the authors have read and complied with the policy of the journal on ethical consent.

## Consent

Written informed consent was obtained from the patient for publication of this case report and accompanying images. A copy of the written consent is available for review by the Editor-in-Chief of this journal on request.

## Provenance and peer review

Not commissioned, externally peer-reviewed.

## Author contribution

Dr MAINASSARA CHEKARAOU Samir designed the concept, analyzed and interpreted the findings, wrote and reviewed the final paper under the supervision of Prof Laila Benjelloun and Prof El Harti Karima.

## Guarantor

Mainassara Chékaraou Samir.

## Registration of research studies

Not appliable

## Declaration of competing Interest

The authors declare that they have no conflict of interest.
